# Comparative Gene Expression Profiling of Benign and Malignant Lesions Reveals Candidate Therapeutic Compounds for Leiomyosarcoma

**DOI:** 10.1155/2012/805614

**Published:** 2012-08-05

**Authors:** Badreddin Edris, Jonathan A. Fletcher, Robert B. West, Matt van de Rijn, Andrew H. Beck

**Affiliations:** ^1^Department of Pathology, Stanford University Medical Center, Stanford, CA 93205, USA; ^2^Department of Genetics, Stanford University School of Medicine, Stanford, CA 94305, USA; ^3^Department of Pathology, Brigham and Women's Hospital, Harvard Medical School, Boston, MA 02115, USA; ^4^Department of Pathology, Beth Israel Deaconess Medical Center, Harvard Medical School, Boston, MA 02115, USA

## Abstract

Leiomyosarcoma (LMS) is a malignant, soft-tissue tumor for which few effective therapies exist. Previously, we showed that there are three molecular subtypes of LMS. Here, we analyzed genes differentially expressed in each of the three LMS subtypes as compared to benign leiomyomas and then used the Connectivity Map (cmap) to calculate enrichment scores for the 1309 cmap drugs in order to identify candidate molecules with the potential to induce a benign, leiomyoma-like phenotype in LMS cells. 11 drugs were selected and tested for their ability to inhibit the growth of three human LMS cell lines. We identified two drugs with *in vitro* efficacy against LMS, one of which had a strongly negative enrichment score (Cantharidin) and the other of which had a strongly positive enrichment score (MG-132). Given MG-132's strong inhibitory effect on LMS cell viability, we hypothesized that LMS cells may be sensitive to treatment with other proteasome inhibitors and demonstrated that bortezomib, a clinically-approved proteasome inhibitor not included in the original cmap screen, potently inhibited the viability of the LMS cell lines. These findings suggest that systematically linking LMS subtype-specific expression signatures with drug-associated expression profiles represents a promising approach for the identification of new drugs for LMS.

## 1. Introduction

Leiomyosarcoma (LMS) is a malignant neoplasm of smooth muscle that accounts for approximately one quarter of all soft-tissue sarcomas. Most frequently, LMS occurs in the uterus or the retroperitoneum, but these tumors can also present in a number of soft tissues throughout the body. Current treatment protocols for LMS consist of surgery with adjuvant doxorubicin-based chemotherapy [1]. There are no effective targeted therapies available for this cancer. Previously, using gene expression profiling, array comparative hybridization, and immunohistochemistry, we identified three distinct biologic subtypes of LMS [2]. The presence of distinct biologic disease subtypes suggests that LMS subtypes may show differential drug responses.

Leiomyoma (LM) is a benign smooth muscle neoplasm that, like its malignant counterpart LMS, frequently occurs in the uterus. While LM is a significant cause of hospitalizations for gynecological disorders and is the most frequent reason for hysterectomies among US women, these growths virtually never metastasize [3]. We hypothesize that genes differentially expressed between LMS subtypes and LM may provide insight into biological pathways driving malignant behavior in LMS and may facilitate identification of drugs to target oncogenic pathways in LMS subtypes.

**Figure 1:**
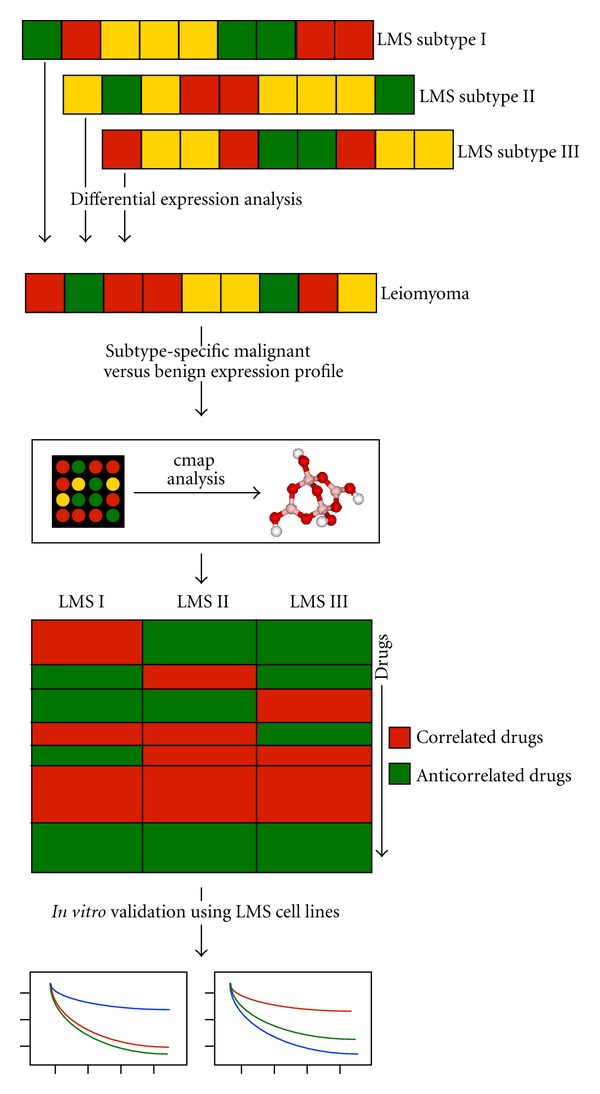
Overview of analytical and experimental workflow. Gene expression profiles from each of the three LMS subtypes were compared to gene expression profiles from LM to identify the top 100 “up” and top 100 “down” differentially expressed genes for each subtype compared with LM. These three pairs of gene lists were then uploaded to cmap to estimate cmap enrichment scores linking drugs with LMS subtype expression signatures. 11 drugs, with a range of cmap enrichment scores, were then tested against three LMS cell lines using *in vitro* drug response experiments.

Here, we aimed to identify and validate new therapeutic molecules for LMS. To do so, we identified genes that were most highly differentially expressed between LM and each of the three LMS subtypes. We then correlated these expression profiles with the Connectivity Map (cmap), a reference collection of gene expression profiles from cultured human cell lines (breast cancer epithelial cell line MCF7, prostate cancer cells PC3, leukemia cells HL60, and melanoma cells SKMEL5) treated with a large and diverse library of small molecules [4, 5]. Previous studies in cancer used cmap to identify drugs with highly negative enrichment scores with cancer signatures, hypothesizing that these molecules were the most likely to show therapeutic efficacy in the cancer type [6–8]. Similarly, we used cmap to generate enrichment scores to indicate the direction and magnitude of the similarity between each LMS subtype expression signature and each drug's effect on gene expression in cancer cell lines. After generating enrichment scores for each drug in cmap with each of the three LMS subtype signatures, we selected 11 drugs (representing a range of enrichment scores) and evaluated each drug's ability to inhibit the viability of three human LMS cell lines *in vitro *(Figure 1). 

## 2. Results

**LMS Subtype Signatures**

To generate gene signatures of each LMS subtype, we performed Significance Analysis of Microarrays [9] and identified the top 100 gene expression features overexpressed in each LMS subtype and the top 100 gene expression features underexpressed in each subtype compared with 19 leiomyoma samples. The gene expression features were mapped to Affymetrix probe IDs, and features without probe IDs were excluded from the subsequent analysis.

**Prediction of Novel Drugs for LMS**

To identify drugs to target the different LMS subtypes, we utilized the Connectivity Map (cmap, http://www.broad.mit.edu/cmap/), which is a publically available resource designed to find connections between disease-associated gene expression signatures and drug response signatures [4, 5]. We uploaded the LMS subtype gene signatures to cmap and generated enrichment scores for each of the 1309 “perturbagens” in cmap. A perturbagen is an agent (small molecule, genetic reagent, etc.) that can be used to produce gene expression changes in cell lines. The enrichment score is a value between +1 and −1, and a high positive score indicates that the perturbagen tended to induce the expression of the query LMS subtype signature, while a high negative score indicates the perturbagen tended to reverse the expression of the query LMS subtype signature. Additional description of the cmap procedure can be found in the methods and are discussed in greater detail elsewhere [4].

**Figure 2:**
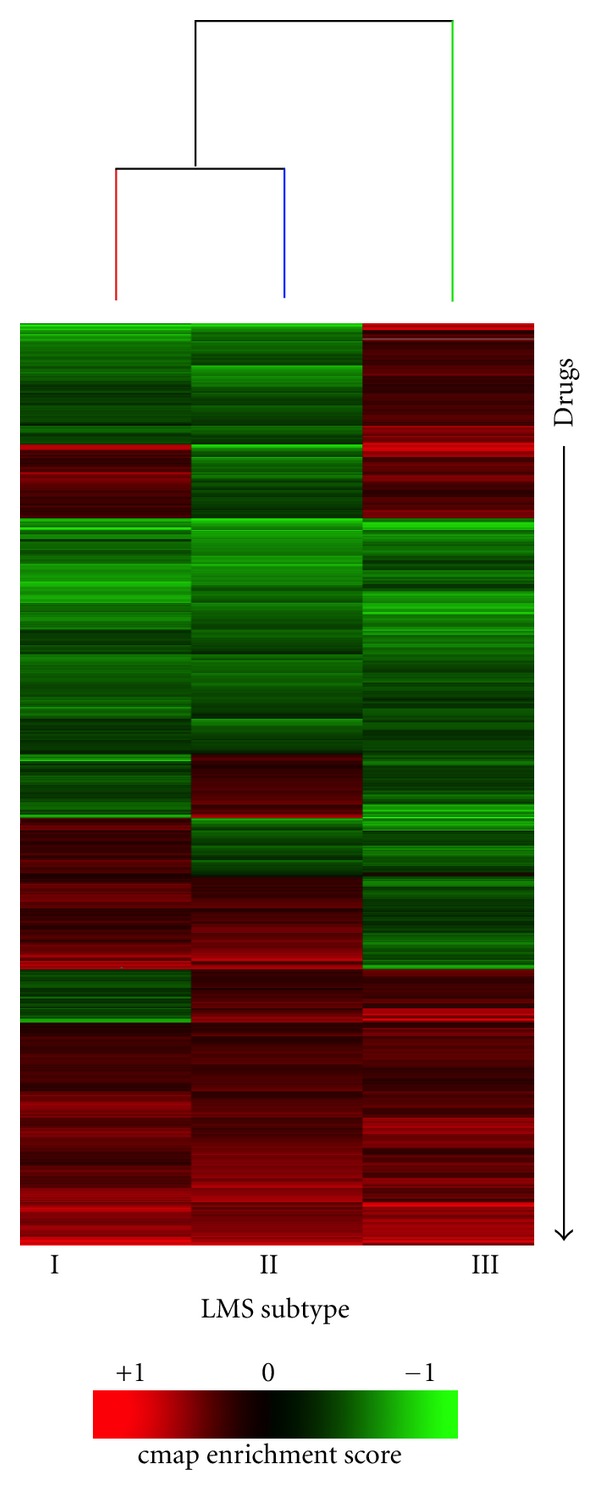
Connectivity Map to identify drugs to target leiomyosarcoma subtypes. Unsupervised hierarchical clustering of the drugs (along the *y*-axis) and three LMS subtypes (along the *x*-axis). The heatmap displays the enrichment score of each drug with each LMS subtype. Green indicates negative enrichment and red indicates positive enrichment. We used these data to test whether the direction and/or magnitude of cmap enrichment scores could predict drug response in LMS cell lines. Full results from the Connectivity Map analysis are provided in Supplemental Table  S4.

This analysis demonstrated that the perturbagens showed highly variable connectivity with the 3 LMS subtypes (Figure 2, Supplemental Table  S4 of the supplementary material available online at doi:10.1155/2012/805614). Cantharidin showed the strongest negative enrichment scores across the three LMS subtypes. Cantharidin is a drug from traditional Chinese medicine, which has been predicted to have anticancer activities through its activity as a protein phosphatase inhibitor [10–12].

The top ranking drugs with the most negative enrichment scores in LMS Subtypes I and II included inhibitors of known oncogenic pathways: Tyrphostin AG-825, a selective tyrosine kinase inhibitor preferentially inhibiting HER-2/neu, and gefitinib, an EGFR inhibitor. The top-ranking drug with the most negative enrichment scores in LMS Subtype III was MG132, a proteasome inhibitor [13]. MG132 achieved a high positive score in LMS Subtype II.

### 2.1. Subtyping LMS Cell Lines

**Figure 3:**
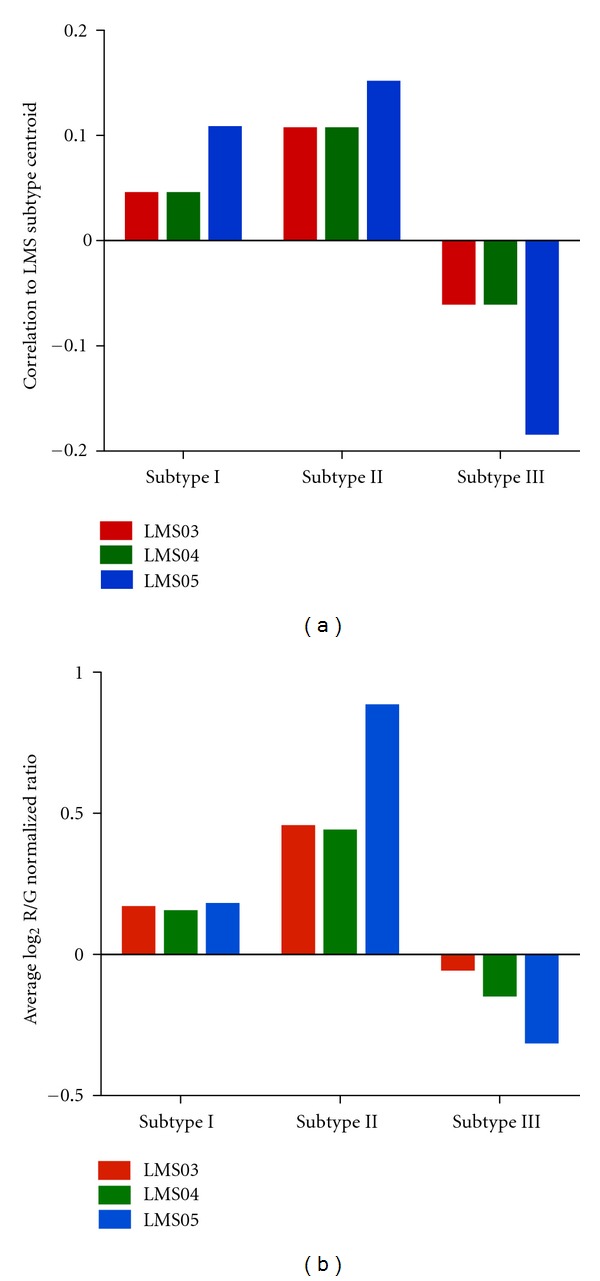
Subtype specificity of LMS cell lines. The correlations of LMS03, LMS04, and LMS05 cell lines to the centroids of the three LMS subtypes were assessed by nearest centroid analysis (a). The average expression levels of LMS subtype I, II, and III genes were evaluated in the three LMS cell lines to identify which LMS subtype these cell lines most closely resembled (b).

We first evaluated the similarity of the LMS03, LMS04, and LMS05 cell lines to expression profiles of the previously defined LMS subtypes using a nearest centroid analysis, in which we computed gene expression centroids for each of the 3 LMS subtypes. We found that all 3 cell lines most closely resembled the LMS subtype II centroid (Figure 3(a)). We next evaluated the mRNA expression levels of previously defined LMS subtype I-, II-, and III-specific genes in the three LMS cell lines. Similar to our nearest centroid analysis results, all three LMS cell lines showed a higher average expression of genes associated with LMS subtype II in comparison to genes associated with LMS subtypes I and III (Figure 3(b)).

### 2.2. Experimental Validation of Cmap Drugs

**Figure 4:**
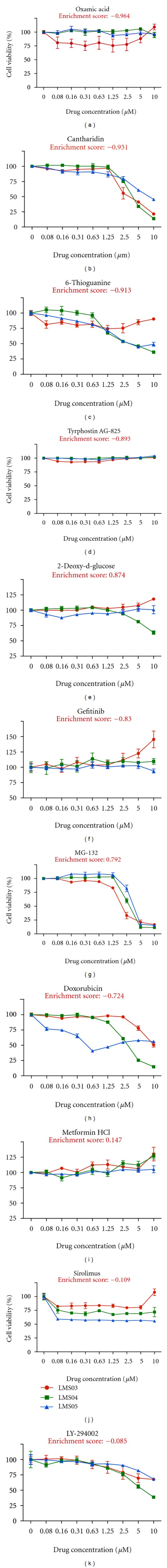
*In vitro* cell viability assays of 11 drugs on three LMS cell lines. Three LMS cell lines, LMS03, LMS04, and LMS05, were exposed to the indicated drugs at concentrations ranging from 0 to10 *μ*M. Cell viability was assessed 72 h after exposure to drugs using WST-1 assays and experiments were performed in triplicate. Data are arranged in order of decreasing magnitude of cmap enrichment scores for LMS Subtype II, which all three cell lines most closely resembled according to our analysis.

We next sought to functionally validate our cmap predictions for novel LMS drugs by performing *in vitro* cell viability assays using three human LMS cell lines, LMS03, LMS04, and LMS05. First, we selected 11 commercially available drugs with cmap enrichment scores to an LMS subtype ranging from approximately −1 to 1 (Table 1). While these drugs do represent a range of enrichment scores, the set of drugs evaluated was highly enriched for drugs with enrichment scores near 1 or −1 (Median of absolute value of enrichment score for 1309 perturbagens in cmap = 0.4 versus 0.79 for the 11 drugs evaluated; Wilcoxon *P* = 0.00009 (Supplemental Figure 1)). Next, we treated the LMS cell lines with each drug for 72 hours, using a concentration range of 0 *μ*M to 10 *μ*M, before assessing cell viability (Figure 4). While most drugs failed to potently inhibit cell viability in all three LMS cell lines, both Cantharidin and MG132 demonstrated strong antigrowth effects across LMS03, LMS04, and LMS05 (Figures 4(b) and 4(j)).

**Figure 5:**
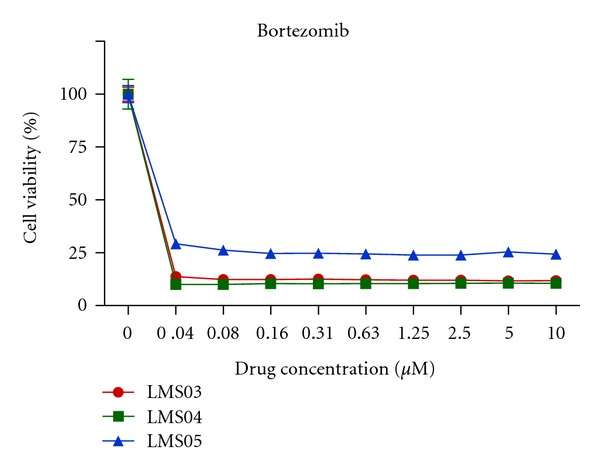
*In vitro* cell viability of LMS cell lines treated with bortezomib. LMS03, LMS04, and LMS05 were exposed to bortezomib at concentrations ranging from 0 to10 *μ*M. Cell viability was assessed 72 h after exposure to drugs using WST-1 assays and experiments were performed in triplicate.

Given MG132's strong inhibitory effect on LMS cell viability, we hypothesized that LMS cell lines may be sensitive to treatment with other proteasome inhibitors. Therefore, we evaluated the antigrowth effects of bortezomib, a clinically approved proteasome inhibitor that was not included in the original cmap screen. After 72 hours of treatment using a concentration range of 0 *μ*M to 10 *μ*M, we found that bortezomib could inhibit the viability of all three LMS cell lines at doses as low as 0.04 *μ*M (Figure 5).

### 2.3. Evaluating Relationship between Cmap Enrichment Score and LMS Drug Response

To determine whether cmap enrichment scores were associated with *in vitro* drug response as measured by LMS cell line viability, we calculated the Pearson correlation between the percent inhibition of cell viability at 10 *μ*M in LMS03, LMS04, and LMS05 for each drug and the cmap enrichment scores determined for all three LMS subtypes. Regardless of the cmap enrichment scores used (subtype I, II, or III), we found no statistically significant association between *in vitro* drug response and cmap enrichment scores (Supplemental Table  S1). To ascertain whether the magnitude of cmap enrichment, and not the positivity or negativity of the score, was predictive of *in vitro* drug response, we similarly calculated the correlation between cell viability and cmap scores, this time using only the absolute value of the cmap enrichment scores. Similarly, we found no statistically significant overall association between the magnitude of a drug's cmap enrichment score and its ability to inhibit *in vitro* LMS cell viability (Supplemental Table  S2).

To specifically evaluate whether highly nonzero enrichment (absolute value >0.75) were associated with *in vitro* drug response as a binary variable, we performed a Fisher's exact test where a significant cmap enrichment score was defined as having an absolute value greater than 0.75 and a significant drug response was defined as having greater than 50% cell viability inhibition at a 10 *μ*M concentration. While we found no statistically significant association between a significant cmap enrichment score and inhibition of cell viability, our data did show a trend suggesting that a highly nonzero cmap enrichment score to LMS subtype II is more likely to be predictive of *in vitro* drug response (8/21, 38% of highly nonzero enrichment scores showed high levels of cell viability inhibition compared with 2/12, 17% of enrichment scores near zero (*P* = 0.26); Supplemental Table  S3).

These findings suggest that further work that would include a larger number of observations is needed to systematically evaluate the relationship between cmap enrichment score and *in vitro* drug response in LMS. However, we note that we selected a set of 11 drugs highly enriched for enrichment scores near 1 or −1, and we did identify 2 of 11 drugs with strong* in vitro *activity. These drugs represent novel candidate drugs for treatment of LMS.

## 3. Discussion

There are currently no targeted therapies available for the treatment of LMS. Treatment of LMS typically consists of surgery with doxorubicin-based chemotherapy and consideration for adjuvant ifosfamide and radiotherapy in selected cases. Doxorubicin-based therapy has only shown a marginal association with improved overall survival, thereby making it important to evaluate additional therapeutic molecules for the treatment of these tumors [1].

Previously, we have shown that there exist three distinct subtypes of LMS, characterized by unique genomic, transcriptional, and protein expression characteristics [2] and we hypothesized that the differences inherent to these tumor subtypes may underlie the heterogeneity in drug responses observed in LMS patients. Previously, gene expression profiles had been shown to be predictive of metastatic outcome in LMS, suggesting that evaluating the transcriptional features of these tumors may provide important insights into the biology of LMS [14]. In the present work, we performed a comparative gene expression profiling study between samples from each of these three LMS subtypes and a set of benign leiomyomas, and we used this analysis to identify drugs predicted to turn each of the LMS subtypes from a “malignant” to a “benign” state.

Of the 11 small molecules evaluated experimentally in our current study, two drugs, Cantharidin and MG-132, were able to strongly inhibit cell viability in all three LMS cell lines tested. Cantharidin is an ancient Chinese medicine that has been demonstrated to have anticancer activity through its inhibition of protein phosphatases [10–12]. Interestingly, in our functional gene set analysis, we previously found that all three LMS subtypes are highly enriched for phosphoproteins compared to the background full *Homo sapiens* genome [2]. The *in vitro* efficacy demonstrated in the current work may provide a starting point for a more rigorous *in vitro* and *in vivo *exploration of Cantharidin or other phosphatase inhibitors for the treatment of LMS.

MG-132 is a potent inhibitor of the proteasome with an ability to specifically reduce the degradation of ubiquitin-conjugated proteins in mammalian cells [13]. Proteasome inhibitors have demonstrated clinical efficacy in several cancers, as is evidenced by bortezomib's 2003 FDA approval for the treatment of relapsed multiple myeloma and mantle cell lymphoma, and carfilzomib, a next-generation proteasome inhibitor, showing promising results in mid-stage clinical trials [15]. Given the strong effect on cell viability observed with MG-132 in LMS cells, we evaluated whether bortezomib (which was not included in the original cmap screen) could similarly inhibit LMS cell viability and found that the drug had extremely potent antigrowth effects on the three LMS cell lines evaluated. Bortezomib had previously been investigated for the treatment of malignant soft-tissue sarcomas in a 21-patient Phase II clinical trial; while the authors concluded that bortezomib had limited activity as a single agent for the treatment of these cancers, it is interesting to note that only four LMS patients were included in this cohort, and that the single confirmed partial response observed in the study was in an LMS patient [16]. Therefore, it may be worthwhile to further investigate the clinical potential of bortezomib for LMS treatment, either as a single agent or in combination with other molecules with demonstrated antigrowth activity in LMS.

Several reports in the literature have utilized comparative gene expression profiling studies between normal and diseased tissues to identify and validate drugs with therapeutic potential. Two such studies utilized cmap to identify potential therapeutic molecules for neuroblastoma and colorectal cancer [6, 7]. A similar approach was recently used to demonstrate that topiramate, an anticonvulsant used to treat epilepsy, showed therapeutic efficacy in a preclinical model of inflammatory bowel disease [16]. While these studies highlight the potential utility of gene expression-based approaches to help identify a molecule that can be repurposed for novel therapeutic indications, little systematic analysis has been performed to evaluate the relationship between the cmap-derived enrichment scores and the actual responses observed in disease models; previous published reports have focused predominantly on documenting the positive associations discovered with little attention paid to predicted associations that were unable to be validated in follow-up experiments.

In the present work, we show that while using cmap we were able to identify two drugs that potently inhibited LMS cell growth, there was no overall statistically significant association between cmap enrichment scores and actual cell viability inhibition *in vitro* in the 11 drugs that we tested. It is important to note that the primary goal of our analysis was to identify new therapeutic drugs for LMS and not to systematically evaluate cmap, and consequently the set of 11 drugs we selected was significantly enriched for drugs with highly nonzero enrichment scores. This design may have increased our ability to identify effective drugs, but gave us little statistical power to rigorously evaluate associations between drug response and cmap score, as we evaluated few drugs with enrichment scores near zero. The two drugs that showed *in vitro* efficacy showed highly divergent enrichment scores, with Cantharidin showing a highly negative score while MG132 showed a strongly positive score. Although our study was not well-powered to identify significant overall associations between enrichment scores and *in vitro* efficacy in LMS, the rate at which we were able to identify drugs that could inhibit cell viability using cmap (2/11 molecules evaluated, or 18.4%) was higher than similarly designed studies that screened entire chemical libraries without *a priori* predictions of efficacy. For example, Rickardson and colleagues observed a 4.4% hit rate (56/1,266 molecules evaluated) for small molecules that could inhibit myeloma cell line growth, and Zhang and colleagues observed a 0.6% hit rate (16/2,816 molecules evaluated) in a screen of new therapeutic compounds that could inhibit thyroid cancer growth [17, 18]. While our data are far from definitive in answering the important question of cmap's utility for identifying effective therapeutic molecules, they do suggest that algorithmic approaches can be taken to increase the success rate of small molecule screens.

Further, while the gene expression profiles of the three LMS cell lines evaluated showed the strongest similarity to LMS Subtype II, it is likely still necessary to evaluate a broader spectrum of cell lines in order to ascertain whether, and to what degree, our LMS patient tumor subtyping is applicable to immortalized cultured cells. Unfortunately, there exist very few human LMS cell lines available for study, thereby limiting our ability to more rigorously investigate the relationship between subtype specificity and drug response. As more LMS clinical specimens are immortalized and made available to the research community for cell culture studies, it would be informative to ascertain their subtype specificity and to characterize their drug response profiles.

Our findings do suggest that further work is needed to systematically assess the relationship between cmap enrichment scores and drug effects in a variety of cancer cell model systems. It will be valuable for future studies of drug candidates identified using computational approaches to document both the identified candidates that are successfully validated as well as the candidates that do not show efficacy in follow-up experiments.

In conclusion, we utilized gene expression profiles to predict novel drug candidates for LMS, and we functionally tested 11 of these drugs using *in vitro* assays. Our study identified two drugs, Cantharidin and MG-132, that showed strong antigrowth effects in LMS cell lines and that may form the starting point for a more focused evaluation of these and similar drugs for the treatment of LMS.

## 4. Materials and Methods

### 4.1. Gene Expression Profiling and Data Processing

The clinico-pathologic features of the 51 LMS cases have been described previously [2]. The 19 LM samples were reviewed by a senior pathologist specializing in sarcoma diagnosis to confirm diagnosis based on published criteria. Total RNA isolation, RNA labeling, and hybridization to 44 K spotted complementary DNA microarrays were carried out using standard procedures as described previously [2] and microarray data are available for download through the Stanford Microarray Database (http://smd.stanford.edu/) [19]. Significance Analysis of Microarrays (SAM) was performed to identify genes differentially expressed between each of the LMS subtypes and the leiomyoma samples [9]. We then used SAM to determine the top 100 genes upregulated (“upsignature”) or downregulated (“downsignature”) in each of the 3 LMS subtypes compared to the 19 leiomyoma samples; these gene lists were used for subsequent analysis.

### 4.2. Connectivity Map

The “upsignature” and “downsignature” generated for each of the 3 LMS subtypes were uploaded into cmap [4, 5]. In cmap, a “perturbagen” is defined as any modality (small molecule, genetic reagent, etc.) that can be used to treat cells and induce gene expression changes. Each perturbagen can be represented by 1 or multiple instances in cmap. An instance consists of a treatment and control cell-line pair with probe sets ordered by their extent of differential expression between the pair. We focused our analysis on a perturbagen-centered (rather than instance-centered) analysis of the cmap data. We focused our analysis on each perturbagen's enrichment score, which is a measure of the enrichment of the perturbagen's instances' connectivity scores among the ordered list of all connectivity scores. The connectivity score is a value between +1 and −1, and a high positive score indicates that the perturbagen induced the expression of the query LMS subtype signature, while a high negative score indicates the perturbagen reversed the expression of the query LMS subtype signature. For each treatment and control cell-line pair (instance) in cmap, the instance's connectivity score with each LMS subtype signature was determined. The connectivity is determined by computing a Kolmogorov-Smirnov (KS) statistic separately for the up and down components of the LMS subtype query signatures. The connectivity score is set to zero if the up and down KS statistics are in the same direction, otherwise the connectivity score is the KS-up score minus the KS-down score. The enrichment scores, which are based on a perturbagen's connectivity scores, is computed for each perturbagen, and the enrichment score values are normalized to the scale −1 to +1. cmap does provide permutation-based *P* values to estimate the probability of observing a perturbagen's enrichment score due to chance; however, only a minority of perturbagens in the database contained nonnull *P* values, due to insufficient replicates, less than 50% nonnull connectivity scores for a perturbagen's instances, or a mean connectivity score of zero. To allow us to use the full database of perturbagens to generate hypotheses, we focused our analysis on perturbagen enrichment scores and not the perturbagen *P* values.

### 4.3. Cell Culture

LMS03, LMS04, and LMS05 cell lines were derived from human LMS clinical specimens and have been described previously [20, 21]. LMS03, LMS04, and LMS05 were grown in RPMI-1640 media (Invitrogen) supplemented with 10% Fetal Bovine Serum (Sigma-Aldrich) and 1% L-glut-Pen-Strep (Gemini Bio-Products). Cells were maintained at 37°C and 5%  CO_2_ and the medium was replaced every 2-3 days.

### 4.4. Drug Preparations

Doxorubicin, Cantharadin, tyrophostin, 6-thioguanine, oxamic acid, 2-Deoxy-d-glucose, and DMOG were purchased from Sigma-Aldrich. MG132 was purchased from AG Scientific. Gefitinib and Metformin HCl were purchased from Tocris Bioscience. LY294002 was purchased from Cell Signaling Technology. Sirolimus was purchased from Cayman Chemical. Bortezomib was purchased from Selleck Chemicals. All drugs were reconstituted in DMSO (Sigma-Aldrich) to create stock concentrations of 10 mM.

### 4.5. Cell Viability Assays

Cells were seeded at a density of 4,000 cells per well in clear 96-well plates (Techno Plastic Products, Trasadingen, Switzerland), incubated for 24 h to allow adherence to the surface, and then treated in quadriplicate for 72 h with nine-point dilution series of the compounds. For all drugs, a concentration range of 0 to 10 *μ*M was used. Cell viability was determined using the WST-1 Cell Proliferation Assay (Roche Diagnostics) according to manufacturer's protocols; average signals were plotted.

### 4.6. Statistical Analysis

For each of the 3 LMS cell lines, we computed the Pearson correlation between the drug's percent inhibition and the drug's enrichment score. To assess whether the magnitude (and not direction) of the score was associated with inhibition, we used the absolute value of the enrichment score in the correlation analysis. To assess whether a discretized enrichment score showed a significant association with discretized cell viability, we discretized the values at a threshold (0.75 for enrichment score, 50% viability at a 10 uM drug concentration for cell viability) and computed a Fisher's exact test.

## Supplementary Material

Supplemental Figure 1: Boxplot of distribution of absolute value of cmap enrichment scores in the 1309 perturbagen's in cmap (left side) vs. the 11 drugs evaluated in LMS cell line (right side). Although the evaluated drug's scores range from *∼*0 to *∼*1, they are enriched for drugs with higher absolute scores.
Supplemental Table S1: Correlation between cmap scores and percent cell viability.
Supplemental Table S2: Correlation between magnitude (absolute value) of cmap scores and percent cell viability.
Supplemental Table S3: Fisher's exact test 2x2 contingency table.
Supplemental Table S4: Full results of the Connectivity Map analysis.

## Figures and Tables

**Table 1:** Mechanisms of action and cmap enrichment scores for drugs tested in LMS cell lines.

Drug name	Mechanism of action	Clinical use	LMS I cmap score	LMS II cmap score	LMS III cmap score
2-Deoxy-d-glucose	Glycolysis inhibitor	Epilepsy, optical imaging agent	0.952	0.874	0.514
MG-132	Proteasome inhibitor, JNK1 activator, and NF-*κ*B inhibitor	None	−0.535	0.792	−0.981
Metformin HCl	Gluconeogenesis inhibitor, AMPK activator	Type II diabetes, gestational diabetes, and polycystic ovary syndrome	−0.217	0.147	0.315
LY-294002	PI3K inhibitor	None	−0.157	−0.085	−0.3
Sirolimus	mTOR inhibitor	Organ transplant rejection	0.135	−0.109	−0.155
Doxorubicin	Topoisomerase II inhibitor	Solid cancers, hematological cancers	−0.536	−0.724	0.409
Gefitinib	EGFR inhibitor	Nonsmall cell lung cancer	−0.941	−0.83	.736
Tyrphostin AG-825	EGFR inhibitor	None	−0.982	−0.893	0.905
6-Thioguanine	Antimetabolite	Acute leukemia, chronic myeloid leukemia	0.564	−0.913	0.866
Cantharidin	Protein phosphatase inhibitor	None	−0.977	−0.931	−0.98
Oxamic acid	Lactate dehydrogenase inhibitor	None	−0.867	−0.964	0.893
