# Supplementary material for: Comparative Gene Expression Profiling of Benign and Malignant Lesions Reveals Candidate Therapeutic Compounds for Leiomyosarcoma
Source: Sarcoma. 2012 Aug 5;2012:805614. doi: 10.1155/2012/805614 (PMC3420093; doi:10.1155/2012/805614)
Supplement: Supplementary file 1 — Supplemental Figure 1: Boxplot of distribution of absolute value of cmap enrichment scores in the 1309 perturbagen's in cmap (left side) vs. the 11 drugs evaluated in LMS cell line (right side). Although the evaluated drug's scores range from ∼0 to ∼1, they are enriched for drugs with higher absolute scores. Supplemental Table S1: Correlation between cmap scores and percent cell viability. Supplemental Table S2: Correlation between magnitude (absolute value) of cmap scores and percent cell viability. Supplemental Table S3: Fisher's exact test 2x2 contingency table. Supplemental Table S4: Full results of the Connectivity Map analysis. [file 805614.f1.zip › Supplemental Figure 1.pdf]

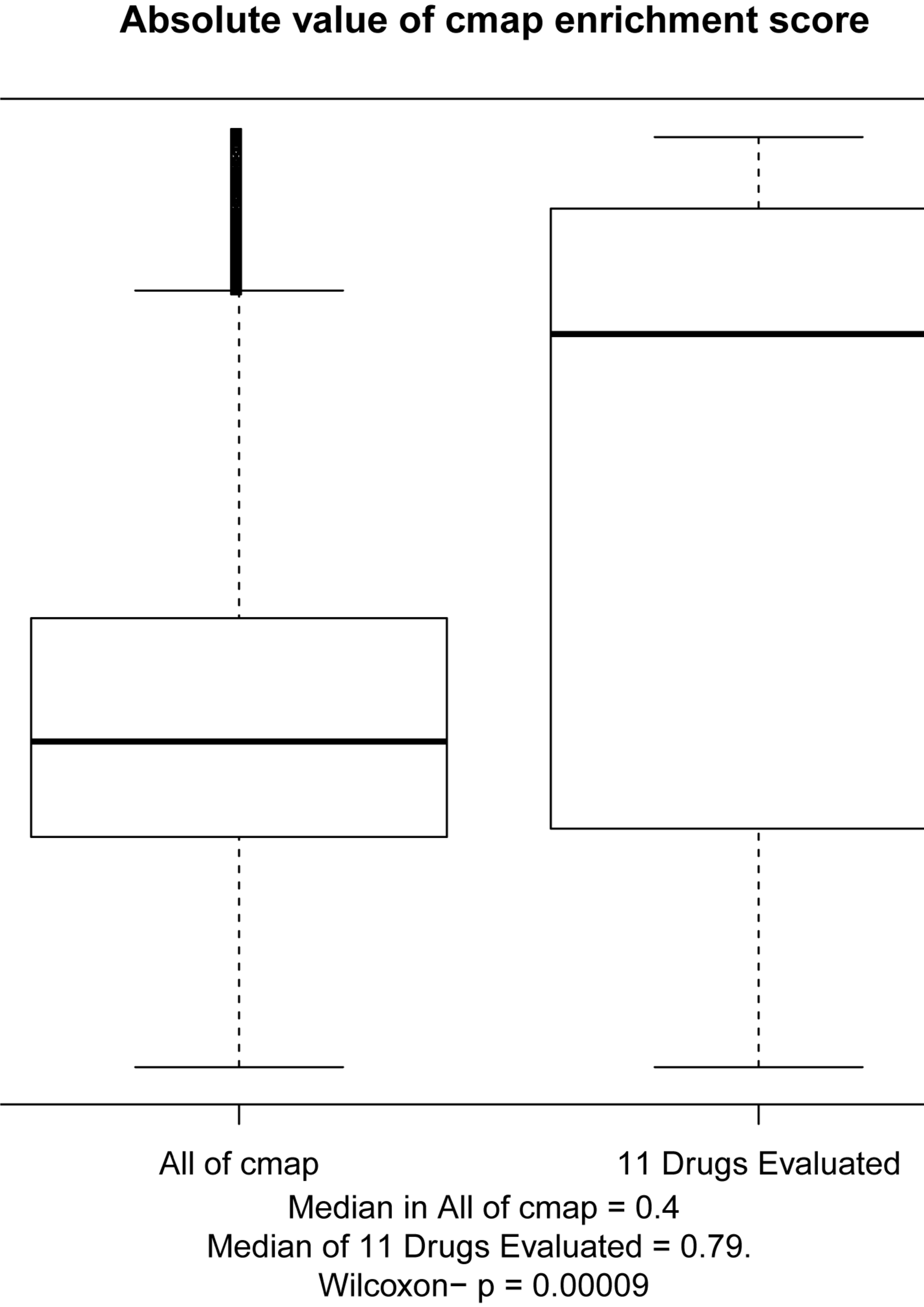
Absolute value of cmap enrichment score
All of cmap
11 Drugs Evaluated
Median in All of cmap = 0.4
Median of 11 Drugs Evaluated = 0.79.
Wilcoxon− p = 0.00009
